# Gunshot bullet embolus with pellet migration from the left brachiocephalic vein to the right ventricle: a case report

**DOI:** 10.1186/1757-7241-18-36

**Published:** 2010-06-20

**Authors:** Nicholas Greaves

**Affiliations:** 1 University Hospitals of Coventry and Warwickshire (Walsgrave site), Clifford Bridge Road, Coventry, UK

## Abstract

We report the case of a 16 year old male who was the victim of a drive by shooting sustaining the rare but recognised complication of cardiovascular bullet embolism. He was seen as a trauma call in the emergency department and CT scanning revealed 70 shotgun pellets scattered throughout left sided sub-cutaneous tissues of the head and neck, and more significantly a single pellet within the right atrium. It is believed to have got there via injury to the left brachiocephalic vein which was demonstrated by extravasation of contrast on the CT scan. He remained stable throughout admission and the injury was managed conservatively. Serial scanning showed the pellet had subsequently migrated into the right ventricle where it has remained since, presumably having become epithelialised. This case report highlights the importance of repeated scanning for the possibility of projectile migration within the cardiovascular system in similar cases of penetrating injury.

## Introduction

The diagnosis and management of penetrating wounds of the great vessels continues to be a major surgical challenge. Their presentation varies from moribund patients to completely stable ones in whom the diagnosis is often missed unless subtle clues are noted [1]. This case study documents the conservative management of a patient who developed a venous bullet embolus after being shot with a shotgun. We aim to review some of the literature on bullet emboli to raise awareness of their existence, investigation and management. With the amount of gun crime increasing the likelihood of seeing such a case is higher. Such a complication can have significant morbidity and mortality unless detected early. Written informed consent was obtained from the patient for publication of this case report.

## Case Report

**Figure 1:**
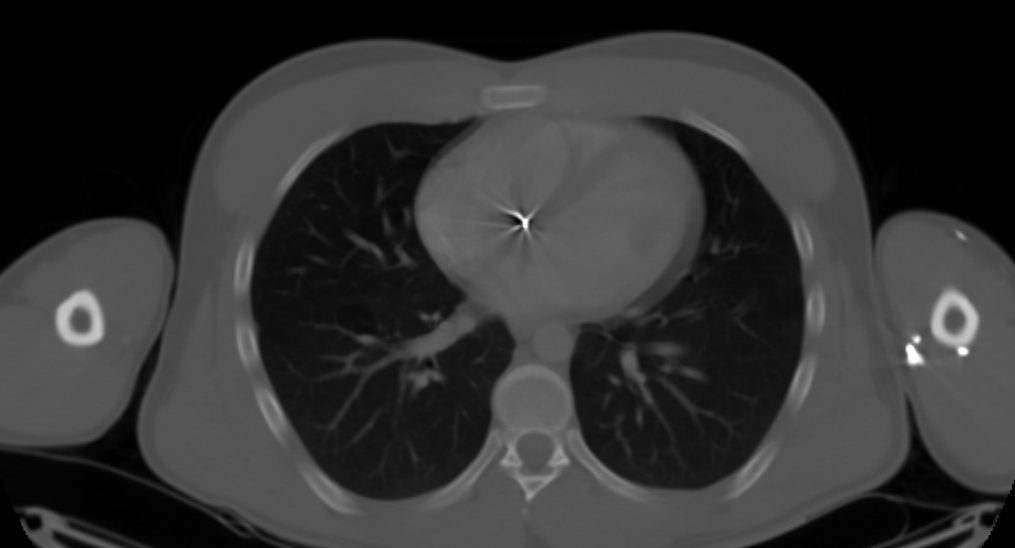
CT thorax viewed with bony windows demonstrating the foreign body (pellet) artefact in the right atrium. Note also the pellets in the left arm.

A 16 year old male presented to our emergency department having been the victim of a 'drive-by' shooting. He was haemodynamically stable with shotgun wounds to the left side of the head and neck. Primary and secondary surveys were essentially normal barring superficial wounds but a CT trauma series was performed to look for occult injuries and establish pellet trajectories. The report confirmed over 70 lead density pellets scattered throughout the sub-cutaneous tissues of the left head, neck and shoulder. It also revealed a single pellet in the right atrium in the absence of any cardiac or mediastinal injury (see figure 1). However, there was evidence of damage to the left brachiocephalic vein with extravasation of contrast. The most plausible explanation for the intra-cardiac pellet was intra-vascular migration from the left brachiocephalic vein to the heart via the superior vena cava.

Given the nature of his injuries the patient was moved to HDU for observation and on day 2 went to theatre for debridement and exploration of neck and facial wounds. Conservative management with antibiotics and serial scanning to monitor further bullet migration was favoured over surgical extraction for the intra-cardiac pellet. This decision was based on the patient being asymptomatic, the pellet being in the right side of the heart and clinical experience of previous similar cases.

**Figure 2:**
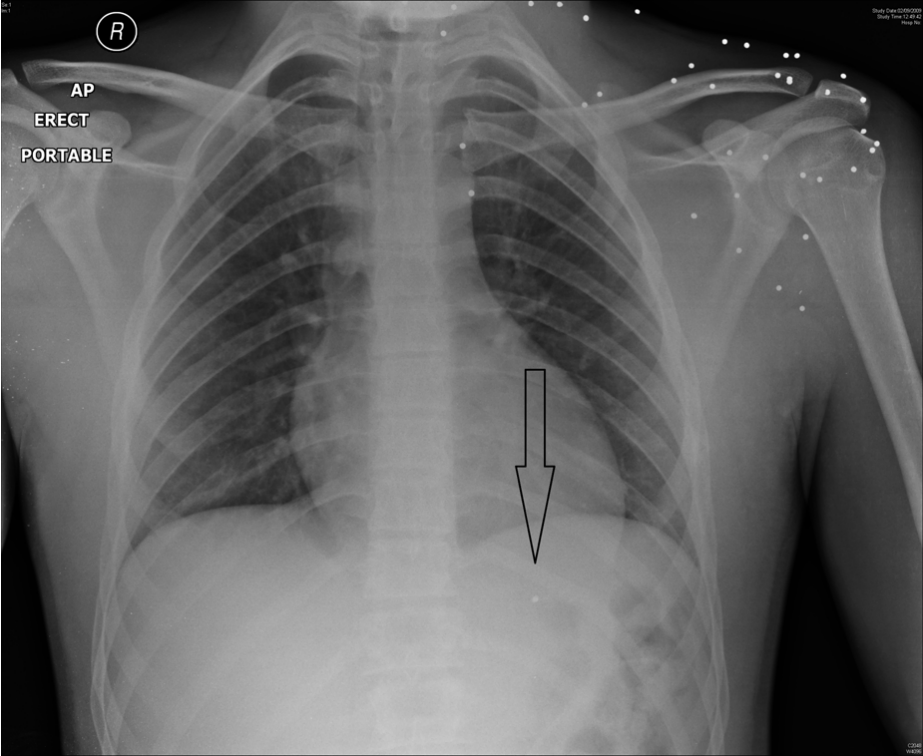
Chest x-ray with arrow demonstrating pellet in right ventricle and multiple pellets in subcutaneous tissue of left shoulder and neck.

A repeat CT scan the following day demonstrated that the pellet had migrated into the right ventricle. A transthroracic echocardiogram on day 3 confirmed normal ventricular and valvular function with the pellet still in the right ventricle. It showed there was no patent foramen ovale or vetriculoseptal defect thereby excluding a paradoxical embolus. The patient remained asymptomatic throughout the admission and was discharged after 4 days. Out-patient review with x-rays at 6 weeks and 6 months after discharge showed the pellet remained within the wall of the right ventricle (see figure 2). He has now been discharged from follow-up.

## Discussion

First described in 1834, foreign body embolisation is a rare complication of penetrating wounds with bullets being the commonest artefact with a quoted incidence of 0.3% [2-5]. A bullet embolus should be suspected in any patient who has a gunshot wound without an exit wound, when the signs and symptoms do not correlate with those expected from the suspected course of the missile and when radiological investigations show that missile location deviates from the path of penetration [6].

Bullet emboli access the vascular system by direct propulsion or erosion into the vessel lumen. 80% are arterial in nature with only 20% being venous, therefore emphasising the rare nature of our case report [7]. Arterial embolisation is symptomatic (claudication, peripheral ischaemia, thrombophlebitis) in 80% of cases [8], and typically originates from the pulmonary artery, heart or great vessels with embolisation to peripheral vessels causing limb ischaemia particularly in the lower extremities [8]. Venous embolisation is symptomatic (dyspnoea, haemoptysis, chest pain) in 30% of cases [9], with embolisation from the large peripheral veins, vena cava or liver, to the right side of the heart, particularly the right ventricle or pulmonary arteries [8,10].

There are 2 rare documented sub-groups of embolisation [1,2]. First is retrograde embolisation seen in 15% of venous cases and defined as projectile movement against the normal direction of blood flow [5,9]. Second is paradoxical embolisation, defined as the passage of a foreign body from the venous to the arterial system by communication through a right to left shunt. Causes include arteriovenous fistula, atrioventricular perforation, ventricular septal defect or patent foramen ovale [2,4,10]. Diagnosis of foreign body emboli is through x-ray, computerised tomography and echocardiography.

Treatment of emboli is controversial. Documented complications of retained intravascular emboli include claudication, parasthesiae, pain, pleural effusion, pericardial effusion, pulmonary abscess, pulmonary infarction, gangrene, endocarditis, arrhythmias, sepsis and cerebral infarction [2,10]. One study reviewing 100 cases found 25% of subjects had embolus related complications with 6% mortality [2]. Given the low complication rate of removal surgery (1-2%), this study advocated extraction in most cases [2,5]. However, it did not discriminate between venous and arterial emboli. Clearly arterial embolisation resulting in limb or cerebral ischaemia requires prompt removal [2,6,10]. However, asymptomatic emboli pose a problem. They can be left in situ if extrication is technically difficult but removal should be attempted if there is a high risk of dislodgement, proximal clot development or delayed arterial insufficiency [6]. Asymptomatic lung emboli can be left with no serious sequelae [1]. Reasons for removal of intra-cardiac pellets include avoidance of major venous obstruction, endocarditis, arrhythmias, myocardial irritability, valvular dysfunction and delayed migration [2,6,10]. Despite this most centres favour conservative management unless the patient acutely deteriorates.

Removal options for intra-cardiac emboli include percutaneous transvenous extraction with operative median sternotomy if this fails or is not available [2,3,8].

## Conclusion

Bullet embolism is a well documented but rare complication of penetrating injury. Unless recognised early it can have significant complications. There is still debate over the best management, particularly when patients remain asymptomatic. Arguments for conservative management include avoidance of surgical risk and current evidence showing that the majority of patients have no complications. However, operative removal excludes the possibility of subsequent embolus related life threatening complications. Our case has highlighted the need for regular imaging in all cases.

Clearly there needs to be further research to provide evidence based guidelines or even a scoring system for such cases calculating subsequent risk of embolic complications. This would help differentiate those high risk patients who would benefit from surgery from those low risk patients who could be managed conservatively.

## Competing interests

The authors declare that they have no competing interests.

## References

[B1] Demerkilic U Yilmaz AT Tatar H Ozturk YO Bullet embolism to the pulmonary artery Interact CardioVasc and Thorac Surg 2004 3 356 358 10.1016/j.icvts.2004.02.002 17670259

[B2] Binning HJS Artho GP Vuong PD Evans DC Powell T Venous bullet embolism to the right ventricle Brit J Rad 2007 80 e296 e298 10.1259/bjr/64277826 18065636

[B3] Palmen M Bekkers JA de Jong PL Bogers AJJC Bullet on the Run: Bullet embolism to the right ventricle after abdominal shot gun injury with bowel perforation Surgery Journal 2007 2 2 22 24

[B4] Symbas PN Kourias E Tyras DH Hatcher CR Penetrating wounds of great vessels Ann Surg 1977 179 No 5 757 761 10.1097/00000658-197405000-00031 PMC1356069 17859862

[B5] Cysne E Souza EG Freitas E Machado E Giameroni R Alves LR Texeira AS LaBrunie P Bullet embolism into the Cardiovascular system Tex Heart Inst J 1982 9 No 1 75 79 15226816 PMC341476

[B6] Michelassi F Pietrabissa A Ferrari M Mosca F Vargish T Moosa HH Bullet emboli to the systemic and venous circulation Brit J Surg 1990 77 486 472 April 10.1002/bjs.1800770432 2408175

[B7] Colquhoun IW Jamieson MP Pollock JC Venous bullet embolism: a complication of airgun pellet injuries Scott Med J 1991 36 16 17 2031167 10.1177/003693309103600107

[B8] Schurr M McCord S Croce M Paradoxical bullet embolism: case report and literature review J Trauma 1996 40 1034 1036 10.1097/00005373-199606000-00034 8656462

[B9] Schmelzer V Mendez-Picon G Gervin AS Case report: transthoracic retrograde venous bullet embolisation J Trauma 2003 55 979 981 10.1097/01.TA.0000028835.94166.6814608178

[B10] Patel KR Cortes LE Semel L Sharma PV Clauss RH Bullet embolism Cardiovasc Surg (Torino) 1989 30 584 590 2674155

